# Development of Single Nucleotide Polymorphism (SNP) Markers for Analysis of Population Structure and Invasion Pathway in the Coconut Leaf Beetle *Brontispa longissima* (Gestro) Using Restriction Site-Associated DNA (RAD) Genotyping in Southern China

**DOI:** 10.3390/insects11040230

**Published:** 2020-04-07

**Authors:** Zhiming Chen, Guihua Wang, Min Li, Zhengqiang Peng, Habib Ali, Lina Xu, Geoff M. Gurr, Youming Hou

**Affiliations:** 1 State Key Laboratory of Ecological Pest Control of Fujian-Taiwan Crops, Fujian Agriculture and Forestry University, Fuzhou 350002, China; czmfzciq@163.com (Z.C.); guihuawang1206@163.com (G.W.); habib_ali1417@yahoo.com (H.A.); kunka-lina@hotmail.com (L.X.); 2 Fujian Provincial Key Laboratory of Insect Ecology, College of Plant Protection, Fujian Agriculture and Forestry University, Fuzhou 350002, China; 3 Rongcheng Customs District of China, Fuzhou 350015, China; 4 Technology Center of Fuzhou Customs District, Fuzhou 350000, China; limin_2926@163.com; 5 Environment and Plant Protection Institute, Chinese Academy of Tropical Agricultural Sciences, Haikou 571101, China; lypzhq@163.com; 6 Department of Entomology, University of Agriculture Faisalabd, Sub Campus Depalpur, Okara 56300, Pakistan; 7 Graham Centre, Charles Sturt University, Orange NSW 2800, Australia

**Keywords:** *Brontispa longissima*, restriction site-associated DNA sequencing, SNPs, population structure

## Abstract

To determine population genomic structure through high-throughput sequencing techniques has revolutionized research on non-model organisms. The coconut leaf beetle, *Brontispa longissima* (Gestro), is a widely distributed pest in Southern China. Here, we used restriction site-associated DNA (RAD) genotyping to assess the invasion pathway by detecting and estimating the degree of genetic differentiation among 51 *B. longissima* accessions collected from Southern China. A total of 10,127 SNPs were obtained, the screened single nucleotide polymorphism (SNP) information was used to construct the phylogenetic tree, *F*ST analysis, principal component analysis, and population structure analysis. Genetic structure analysis was used to infer the population structure; the result showed that all accessions were divided into Hainan population and non-Hainan population. The Hainan population remained stable, only the Sansha population differentiated, and the non-Hainan populations have gradually differentiated into smaller sub-populations. We concluded that there are two sources of invasion of *B. longissima* into mainland China: Taiwan and Hainan. With the increase of the invasion time, the Hainan population was relatively stable, and the Taiwan population was differentiated into three sub-populations. Based on the unrooted phylogenetic tree, we infer that Taiwan and Hainan are the two invasive base points. The Taiwan population invaded Fujian, Guangdong, and Guangxi, while the Hainan population invaded Yunnan and Sansha. Our results provide strong evidence for the utility of RAD sequencing (RAD-seq) in population genetics studies, and our generated SNP resource could provide a valuable tool for population genomics studies of *B. longissima* in the future.

## 1. Introduction

The coconut leaf beetle, *Brontispa longissima* (Gestro) (Coleoptera: Chrysomelidae), is a serious pest of the coconut palm *Cocos nucifera* (L.) and other palm trees [1]. The species *longissima* was first described as *Oxycephala longissima* by Gestro(1885), collected from the Aru Islands, which are located in the Arafura Sea between New Guinea Island and Australia, and then was transferred to the genus *Brontispa* by Gestro (1907) [1,2]. The beetle is thought to be native to Indonesia and Papua New Guinea [3]. Shun-Ichiro Takano et al. (2011) showed that *B. longissima* represents two monophyletic clades, using mitochondrial DNA analysis and crosses between the two nominal species. One named Pacific clade is distributed in a relatively limited area (Papua New Guinea, Australia, Samoa, and Sumba Island), whereas the other named Asian clade covers a wide area, including Asia (i.e., Indonesia, Cambodia, Japan, Myanmar, the Philippines, Taiwan, Thailand, and Vietnam) and the French Polynesia, New Caledonia, and Vanuatu [2]. Since the late 1930s, it has invaded Pacific islands [3,4]. In 1975, the beetle was introduced to Taiwan [5], and then spread to Hong Kong, Hainan, Guangdong, Guangxi, Yunnan, and Fujian provinces in China [6]. Larvae and adults of *B. longissima* are found in young folded leaflets of palms, where they feed on the soft leaf tissues [7]. Infestation with the beetles turns the leaves brown and decreases fruit production. The sustained heavy attack may ultimately kill the palm trees [3,7,8]. At present, the prevention and control of *B. longissima* are mainly accomplished via chemical and biological control [9]. Due to the large quantities of pesticides used, chemical methods are not only expensive but can also induce insecticide resistance and pollute the environment. The cost and effects of the biological control methods are not yet obvious, thus a new strategy is needed to directly, safely, and effectively control the damage caused by the *B. longissima*.

Related studies on population genetic structure and genetic diversity identified genetic evolution and gene connectivity between different populations of pest, which has important theoretical and practical significance for comprehensive management [10,11,12]. Population differentiation and the genetic variation of pests directly affects the formulation and application of many environmentally friendly pest control strategies, such as infertility techniques, mating interference techniques, microbial pesticides, etc. Early studies mainly focused on biology, ecology, and morphological classification of *B. longissima*, with few studies that focused on molecular genetics. To date, the use of gene markers to analyze genetic diversity and the genetic structure of *B. longissima* has not been reported. Population genetics research is based on a relatively wide range of distribution [13]. The same species are distributed in a relatively wide range of different geographical environments. Due to factors such as climate and host, microevolution can be accelerated, and more genetic variation can be obtained in a short time. However, highly connected and recently differentiated populations with large, effective population sizes typically exhibit very weak genetic differentiation, reducing the ability of genetic tools for defining management units and assigning accessions to their origin [14]. Therefore, to obtain a high-resolution profile of the population structure, more nuclear and mitochondrial genetic markers are required [15]. To date, the available genetic markers for *B. longissima* have been limited to mitochondrial DNA, restriction fragment length polymorphism (RFLP) [2,16], and microsatellite techniques [4].

The advent of next-generation sequencing (NGS) has facilitated the identification of novel population genetic markers on an unprecedented scale, even in non-model organisms [17]. Restriction site-related DNA sequencing (RAD-seq) is a promising technique widely used in population genomics. In particular, RAD-seq uses a repeatable method to generate a large number of nuclear markers, in which accession single nucleotide polymorphisms (SNPs) detected by short NGS read nearby or between restriction sites scattered throughout the nuclear genome [18]. In contrast to whole genome sequencing, RAD-seq only targets a subset of the genome. This not only improves the sequencing depth of each locus, but also includes more accessions in a single sequencing run [19]. Thus, we hypothesized that genetic diversity of *B. longissima* populations could be useful for the control of coleopterans in other regions of the world.

In this study, we aimed to elucidate the genetic structure of *B. longissima* populations using RAD sequencing and SNP markers. The results of this study have important implications for understanding the genetic diversity of *B. longissima* populations and could be used to guide the integrated control of the beetle.

## 2. Materials and Methods

### 2.1. Sampling

**Figure 1:**
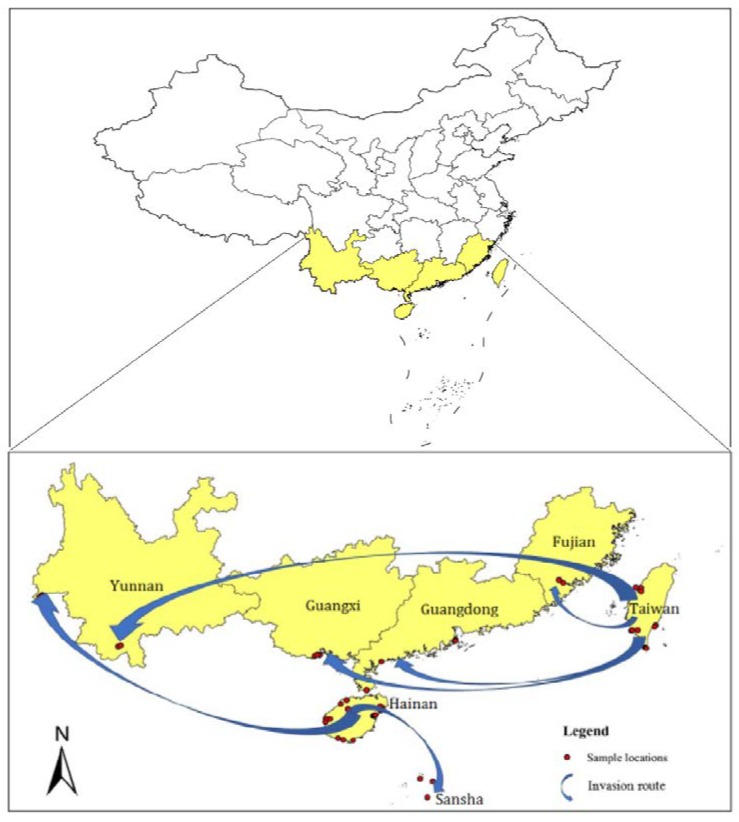
Map of coconut leaf beetle, *B. longissima* sampling locations and inferred invasion routes.

*B. longissima* specimens were collected from May 2016 to August 2017. The sampling range was mainly in Southern China, including the Fujian (FJ), Guangdong (GD), Guangxi (GX), Yunnan (YN), Hainan (HN), Sansha (SS), and Taiwan (TW) regions, as shown in Table 1 and Figure 1. A total of 51 accessions were collected and all were captured on coconut trees *C. nucifera.* Detailed descriptions with the original locations of specimen collection are provided in Table 1. The specimens we sampled were preserved in 95% ethanol (Xilong Scientific, Shantou, Guangdong, China) for DNA extraction. Then, the samples were maintained in 10% ethanol (Zhongshan Scientific, Nanjing, Jiangsu, China) for later identification. Voucher specimens were catalogued for further experimentation in the laboratory.

### 2.2. RAD Library Preparation and Illumina Sequencing

RAD-seq libraries were constructed following a modified protocol [17]. Genomic DNA (0.1–1 μg from accession samples) was extracted, and the restriction endonuclease *EcoR*I (New England Biolabs) was used to digest the genome followed by heat inactivation of the enzyme. The EcoRI-cut site of each sample was ligated with barcoded P1 adapters (complementing the *EcoR*I-cut DNA gap). These adapters contained forward amplification and Illumina sequencing primer sites, as well as a nucleotide barcode 4- or 8-bp long for sample identification. The adapter-ligated fragments were subsequently pooled, randomly sheared, and size-selected. DNA was then ligated to a second adapter (P2), a Y adapter [20] that has divergent ends. The reverse amplification primer was unable to bind to P2 unless the complementary sequence was filled in during the first round of forward elongation originating from the P1 amplification primer. The structure of this adapter ensured that only P1 adapter-ligated RAD tags were amplified during the final PCR amplification step. During the QC (Quality Control) step, Agilent 2100 Bioanaylzer and qPCR methods are used to qualify and quantify the sample library. Then, the library products were used for sequencing. Sequencing was performed on the Illumina HiSeq 4000 platform (Illumina company, San Diego, California, USA).

### 2.3. Clean Reads Filtering and SNP Obtaining

Raw reads of RAD-seq Illumina sequencing were processed using the Stacks pipeline [21,22]. Quality filtering was performed with the process–radtags function implemented in Stacks with default settings [15]. According to the following standards: (1) removing reads aligned to the barcode adapter, (2) remove low quality reads (i.e., reads with more than 50% bases whose quality value is less than or equal to 10), and (3) removing reads with ≥10% unidentified nucleotides. We identified the loci of each sample using the ustacks program in the Stacks, then the loci in all accessions were integrated using the cstacks program to form a catalog. One-to-one searches and probability calculations were performed by the sstacks program for the gene locus appearing in each accession and the gene locus appearing in the catalog, which defined the alleles at each locus of the gene. [23]

The final SNPs obtained underwent strict filtering and screening. We selected only SNPs matching the following criteria: (1) At least 20 accessions contained the locus in which the SNP was located, (2) the) was 0.01, (3) the minimum depth of the gene locus in which the SNP was located was 7, and (4) if all samples had a deletion rate of more than 10% at a site, the site was removed. The Stacks denovo_map.pl was implemented to identify candidate SNP markers for downstream analyses, as no reference genome for *B. longissima* was available.

### 2.4. Population Genetic Relationship Analysis 

#### 2.4.1. Population Structure Analysis

Based on the candidate SNPs, we inferred the population structure of *B. longissima* using Structure 2.3.4 [24,25,26,27]. The statistic “K”, which indicates the change in likelihood of different numbers of clusters, was calculated, and the cluster number with the highest K value, which indicated the most likely number of clusters in the population, was obtained by using Structure Harvester (available at http://taylor0.biology.ucla.edu/structureHarvester/). Based on the K value that we have selected, pairwise *F*ST values were estimated in Arlequin v.3.5 [28,29].

#### 2.4.2. Principal Component Analysis (PCA)

PCA was performed by using PLINK [30,31], without prior information on group accession populations; PCA was performed to visualize broad-scale population structure. 

#### 2.4.3. Clustering of Accessions and Populations

The phylogenetic tree is a branch map or tree that describes the order of evolution between groups, indicating the evolutionary relationship between groups [32]. The theoretical methods for constructing phylogenetic trees are mainly Neighbor-joining (NJ) and Maximum likelihood (ML). ML tree was generated based on the GTR (generalized time-reversible) substitution model using PhyML [33] with 1000 bootstraps. Tree topology (t), branch length (l), and rate parameters (r) were optimized in this analysis. The SNPs selected can be used calculate the distance matrix in phylogenetic tree. By analyzing the phylogenetic tree constructed by the ML method, we could find the evolutionary relationship between populations.

## 3. Results

### 3.1. Selecting Candidate SNPs for Demographic Inference

A genome scan of the 61,182 SNPs retained using ARLEQUIN [34] revealed that 39,735 SNPs had a MAF of < 0.05, and 10,127 had a missing rate of <10%. Subsequent inferences of the genetic structure were conducted using the 10,127 SNP candidate markers. (Table 2)

### 3.2. Population Genetic Relationship Analysis

#### 3.2.1. Population Structure Analysis

**Figure 2:**
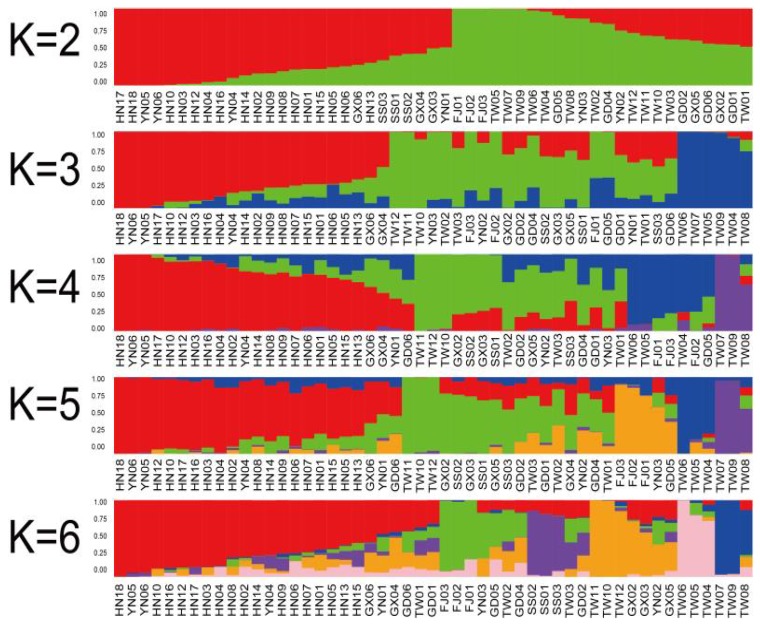
Bayesian plot (K) of group assignment of each accession into 2 to 6 cluster structure analysis of *B. longissima*. The results are grouped by location of sample collections. Each vertical column represents one accession and the coloration is proportional to the accessions estimated membership coefficient in one of the clusters.

**Figure 3:**
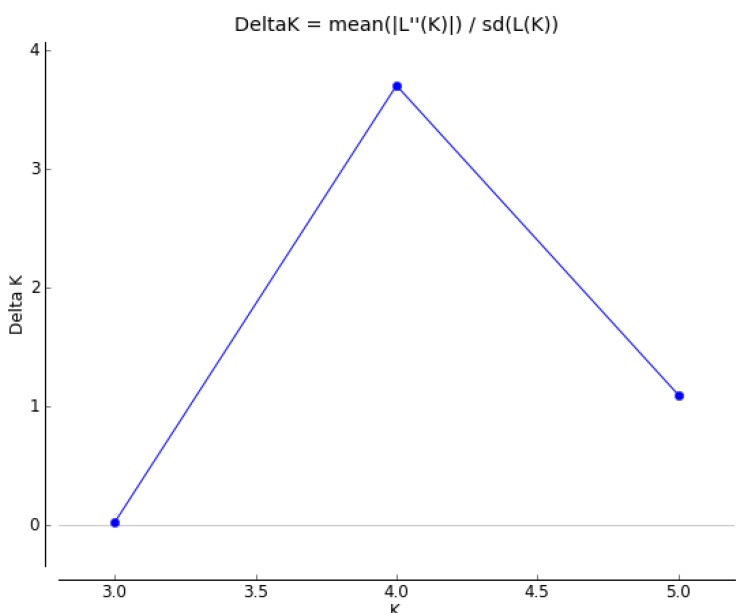
Determination of K using STRUCTURE.

After the stringent filtering procedure, 10,127 loci were identified as candidate SNPs. The candidate SNPs were used for subsequent population inference. The STRUCTURE analysis software was used to analyze the population structure of *B. longissima* (Figure 2). Population structure analysis for each K value was performed, as well as K analysis for a different number of clusters (K) for 51 *B. longissima* accessions. K showed a peak at 4, suggesting four clusters as the optimal option (Figure 3). At K = 2, all accessions are divided into Hainan population and non-Hainan population. From K = 3 to K = 6, the Hainan population remains stable, only the Sansha population is differentiated, and non-Hainan populations have gradually differentiated into smaller subpopulations. With the optimal K = 4, all of the accessions are divided into 4 populations. Population 1 is the Hainan population. Population 2 is the Mainland of Southern China population, including Guangdong, Guangxi, and Yunnan. Population 3 is the Taiwan Strait population, including Fujian and Taiwan. Population 4 is the Taiwan Taichung population, indicating the population differentiation with *B. longissima* accessions collected in Taiwan.

#### 3.2.2. F-statistics

Based on the optimal K = 4, RAD-seq analysis revealed that the overall degree of genetic differentiation among *B. longissima* populations was not high (average pairwise *F_ST_* = 0.093 across all populations) on a reduced-representation genome scale (Table 3). However, the genetic differentiation of population 4 was significantly high (average pairwise *F_ST_* = 0.141). Our result is similar to the genetic differentiation on population structure (Figure 2). The accessions of population 4 were collected from Taichung, Taiwan. We speculated that the high altitude and low temperature environment may have caused the rapid evolution of *B. longissima.*

#### 3.2.3. Principal Component Analysis

**Figure 4:**
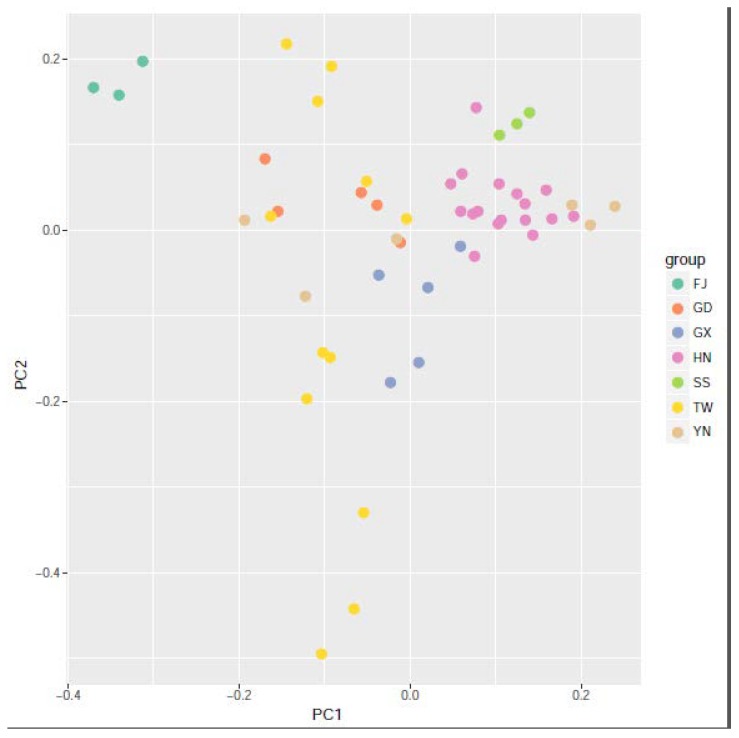
Principal component analysis (PCA) of genetic differentiation among *B. longissima* populations.

The genetic split between different populations was also discerned by PCA in Figure 4. Separation along the first discriminant function (PC1) shows that all accessions are divided into two populations, Hainan population and no-Hainan population, which is consistent with the K = 2 analysis of population structure analysis and indicates the distinct genetic distance between accessions from Hainan and Taiwan. PC2 shows no-Hainan population differentiated into several subgroups. It was consistent with the results of the population structure as shown above.

#### 3.2.4. Phylogenetic Analysis of 51 *B. longissima* Accessions

**Figure 5:**
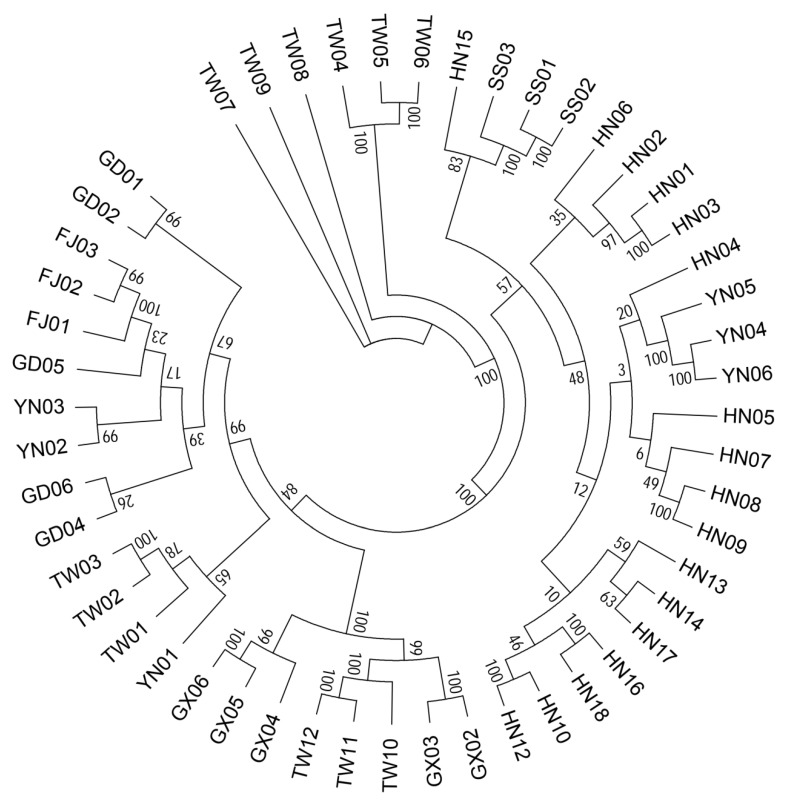
Maximum likelihood (ML) tree of *B. longissima* accessions collected from Southern China.

The filtered SNP information was used to construct a phylogenetic tree using the ML method (Figure 5). The results showed that the accessions collected from Hainan were clustered together, and this cluster was obvious (Figure 2, Figure 4, and Figure 5). *B. longissima* in Fujian was quite different from all other accessions, indicating that the recently distinct population was formed in Fujian. Samples collected in Taiwan were clustered into two major clades, which is consistent with the aforementioned population results, indicating several distinct groups of *B. longissima* detected in Taiwan and mainland China. All *B. longissima* accessions in Hainan, together with accessions from Sansha and Yunan, were clustered together and showed an apparent genetic distance from other accessions which might have originated from Taiwan. 

With a comprehensive analysis of genetic structure, PCA, *F*ST, and the phylogenetic tree, we could indicate the invasion pathway. These results indicated two potential independent populations formed in Taiwan and Hainan, which then invaded mainland China by two independent pathways (Figure 1). 

## 4. Discussion

This study is the first attempt to understand the genetic variation patterns of *B. longissima* in southern China. In this study, we analyzed the genetic diversity and genetic structure among 18 geographic populations collected from 6 provinces in southern China. The use of SNP molecular markers to discover the genetic variability of *B. longissima* showed that its genetic diversity is not related to geographical distance and that it is affected by human factors. According to the analysis of SNP data, the genetic diversity of *B. longissima* is low to medium. 

RAD sequencing is generally used in marine organisms and plant research, to develop SNP molecular markers, but less applied in insect research. The researchers used this method to analyze the genetic structure and genetic diversity of white perch *Morone Americana* [15], Nujiang catfish *Creteuchiloglanis macropterus* [35], American lobster *Homarus americanus* [36], Sweetpotato *Ipomoea batatas* [37], and other organisms. All of them obtained good results. For *B. longissima*, researchers used mtDNA molecular markers [2]. In general mtDNA is a good option for phylogenetic analysis, since it reflects only maternal inheritance and it is not submitted to recombination; thus, its variation is expected to be low within subpopulations of the same species, but higher at species level. In addition, *B. longissima* invaded China for a short time, only about 40 years, and the genetic differentiation within the species was not obvious. SNP markers are highly polymorphic. Thus, these markers should be good markers to estimate the population structure of *B. longissima*.

ML tree shows that samples collected in Taiwan were clustered into two major clades, indicating several distinct groups of *B. longissima* detected in Taiwan and mainland China. All *B. longissima* accessions in Hainan, together with accessions from Yunan, were clustered together and showed apparent genetic distance from other accessions which might have originated from Taiwan. According to the results of the phylogenetic tree analysis, the invasion route of *B. longissima* in southern China can be inferred. Taiwan and Hainan are the two invasive base points. The Taiwan population invades Fujian, Guangdong, and Guangxi, and the Hainan population invades Yunnan and Sansha (Figure 1).

*B. longissima* is thought to be native to Indonesia and Papua New Guinea, Shun-Ichiro Takano et al. (2011) showed that *B. longissima* represents two monophyletic clades, using mitochondrial DNA analysis, and crosses between the two nominal species. One named Pacific clade is distributed in a relatively limited area, whereas the other named Asian clade covers a wide area, including Asia and the French Polynesia, New Caledonia, and Vanuatu [2]. Java Island was the earliest recorded point for the spread of the Asian clade [38,39]. However, the historical data shows that *B. longissima* Asian clade appeared in New Caledonia and Vanuatu [40], Tahiti [41], Taiwan [42], Japan [43], the Indo-China peninsula and the Philippines [44], and Hainan of China. The populations other than China have not been obtained, thus we are not sure about the clade to which that Chinese population belongs and it is the shortcoming of our study.

As an invading pest, *B. longissima* has high risk and great harm. The results showed that most effective measures for comprehensive treatment of *B. longissima* are to strengthen the quarantine work during the transport of *B. longissima* host seedlings to prevent their spread, and then use chemical agents [9] or parasitic bees [45] to kill them in the occurrence area.

## 5. Conclusions

In this study, the RAD sequencing technology was used for the development of *B. longissima* SNP genetic markers. In our strict filtering and screening, 10,127 SNPs out of 61,182 markers were identified. Based on these SNPs, phylogenetic tree analysis, principal component analysis (PCA), and population structure analysis were used to analyze the evolutionary relationship among different *B. longissima* populations. The results showed that the *B. longissima* in China can be divided into Hainan *B. longissima* population and non-Hainan *B. longissima* population. The Hainan *B. longissima* population was relatively stable. The non-Hainan *B. longissima* population was divided into several subgroups. Based on the unrooted phylogenetic tree, we inferred that Taiwan and Hainan *B. longissima* populations evolved as two invasion hubs. The Taiwan *B. longissima* population invaded Fujian, Guangdong, and Guangxi, while the Hainan *B. longissima* population invaded Yunnan and Sansha. However, due to the invasion in China, the time is short, less than 40 years, thus a genetic differentiation is not obvious enough.

## Figures and Tables

**Table 1:** Sampling locations and information of *B. longissima.*

Region	Sampling Location	Code	Coordinates	Altitude (m)
China Mainland	Zhanjiang, Guangdong	GD-01	20°16′7″ N 110°13′26″ E	1
	Zhanjiang, Guangdong	GD-02	21°23′32″ N 110°46′29″ E	0
	Zhuhai, Guangdong	GD-04	22°13′14″ N 113°33′38″ E	1
	Zhuhai, Guangdong	GD-05	22°15′44″ N 113°35′1″ E	1
	Zhuhai, Guangdong	GD-06	22°13′56″ N 113°34′35″ E	1
	Beihai, Guangxi	GX-02	21°24′21″ N 109°8′52″ E	0
	Beihai, Guangxi	GX-03	21°26′59″ N 109°3′4″ E	37
	Fangchenggang, Guangxi	GX-04	21°37′1″ N 108°14′32″ E	5
	Fangchenggang, Guangxi	GX-05	21°39′37″ N 108°26′27″ E	26
	Fangchenggang, Guangxi	GX-06	21°39′58″ N 108°21′7″ E	2
	Xishuangbanna, Yunnan	YN-01	21°59′23″ N 100°47′2″ E	539
	Xishuangbanna, Yunnan	YN-02	21°59′4″ N 100°46′41″ E	541
	Xishuangbanna, Yunnan	YN-03	22°1′40″ N 100°52′34″ E	838
	Ruili, Yunnan	YN-04	24°01′49″ N 97°54′12″ E	773
	Ruili, Yunnan	YN-05	23°57′15″ N 97°47′36″ E	759
	Ruili, Yunnan	YN-06	24°0′1″ N 97°53′17″ E	765
	Zhangzhou, Fujian	FJ-01	24°37′49″ N 117°31′3″ E	25
	Zhangzhou, Fujian	FJ-02	24°37′19″ N 117°31′28” E	25
	Zhangzhou, Fujian	FJ-03	24°29′54″ N 117°40′38″ E	8
Hainan Island	Wenchang, Hainan	HN-01	19°32′48″ N 110°47′49″E	29
	Wenchang, Hainan	HN-02	19°37′43″ N 110°44′4″ E	8
	Wenchang, Hainan	HN-03	19°28′59″ N 110°44′27″ E	3
	Qionghai, Hainan	HN-04	19°14′52′ N 110°27′46″ E	21
	Qionghai, Hainan	HN-05	19°16′55″ N 110°30′12″ E	16
	Qionghai, Hainan	HN-06	19°18′13″ N 110°32′20″ E	29
	Sanya, Hainan	HN-07	18°17′18″ N 109°42′43″ E	5
	Sanya, Hainan	HN-08	18°22′2″ N 109°7′57″ E	11
	Sanya, Hainan	HN-09	18°18′27″ N 109°19′32″ E	12
	Ledong, Hainan	HN-10	18°41′22″ N 108°47′33″ E	48
	Ledong, Hainan	HN-12	18°41′57″ N 108°42′17″ E	9
	Dongfang, Hainan	HN-13	19°7′56″ N 108°49′60″ E	73
	Dongfang, Hainan	HN-14	19°8′10″ N 108°39′57″ E	10
	Dongfang, Hainan	HN-15	19°1′22″ N 108°39′20″ E	13
	Danzhou, Hainan	HN-16	19°30′33″ N 109°30′8″ E	138
	Danzhou, Hainan	HN-17	19°42′29″ N 109°13′50″ E	3
	Danzhou, Hainan	HN-18	19°51′45″ N 109°26′42″ E	20
Taiwan Island	Pingtung, Taiwan	TW-01	22°37′22″ N 120°29′52″ E	2
	Pingtung, Taiwan	TW-02	22°36′16″ N 120°16′18″ E	2
	Pingtung, Taiwan	TW-03	22°37′25″ N 120°17′59″ E	6
	Taitung, Taiwan	TW-04	22°48′41″ N 121°11′39″ E	1
	Taitung, Taiwan	TW-05	22°49′35″ N 121°11′18″ E	0
	Taitung, Taiwan	TW-06	22°45′51″ N 121°9′43″ E	6
	Taichung, Taiwan	TW-07	24°19′27″ N 120°26′35″ E	210
	Taichung, Taiwan	TW-08	24°17′25″ N 120°37′32″ E	305
	Taichung, Taiwan	TW-09	24°10′1″ N 120°38′1″ E	348
	Kenting, Taiwan	TW-10	21°57′19″ N 120°46′12″ E	4
	Kenting, Taiwan	TW-11	21°56′16″ N 120°48′32″ E	5
	Kenting, Taiwan	TW-12	21°55′8″ N 120°50′13″ E	4
Sansha Islands	Xisha	SS-01	16°46′5″ N 112°14′13″ E	0
	Xisha	SS-02	16°39′55″ N 112°44′27″ E	0
	Xisha	SS-03	16°1′52″ N 112°31′13″ E	0

**Table 2:** Number of putative single nucleotide polymorphisms (SNPs) retained following each filtering step.

Basic Information	No. SNP Variants	Notes
Total SNPs	61,182	Total SNPs identified in Stack pipeline
MAF < 0.05	39,735	
Missing rate < 10%	10,127	Filtered SNP for further analysis

MAF—minimum allele frequency

**Table 3:** F-statistics among 51 sampling locations distributed in the southern regions of China. (The color coding of populations corresponds to Figure 2 (K = 4); red represents population 1, green represents population 2, blue represents population 3, and purple represents population 4.).

F_ST_	Population 2	Population 3	Population 4
Population 1	0.035003	0.068543	0.15456
Population 2		0.032398	0.11385
Population 3			0.15444

## Data Availability

*Brontispa longissima* DNA sequences: All raw RAD-seq reads data can be accessed at NCBI SRA. Bioproject # PRJNA613374 (https://dataview.ncbi.nlm.nih.gov/object/PRJNA613374?reviewer=pm0vvg15fmosutc52lekl0frvt).
